# Assessing the nutritional quality of diets of Canadian children and adolescents using the 2014 Health Canada Surveillance Tool Tier System

**DOI:** 10.1186/s12889-016-3038-5

**Published:** 2016-05-10

**Authors:** Mahsa Jessri, Stephanie K. Nishi, Mary R. L’Abbe

**Affiliations:** Department of Nutritional Sciences, Faculty of Medicine, University of Toronto, FitzGerald Building, 150 College Street, Toronto, ON M5S 3E2 Canada; Clinical Nutrition & Risk Factor Modification Center, St. Michael’s Hospital Toronto, Toronto, Canada

**Keywords:** Nutrient profiling, 2014 Health Canada Surveillance Tool Tier system, Children, Adolescents, Canadian

## Abstract

**Background:**

Health Canada’s Surveillance Tool (HCST) Tier System was developed in 2014 with the aim of assessing the adherence of dietary intakes with Eating Well with Canada’s Food Guide (EWCFG). HCST uses a Tier system to categorize all foods into one of four Tiers based on thresholds for total fat, saturated fat, sodium, and sugar, with Tier 4 reflecting the unhealthiest and Tier 1 the healthiest foods. This study presents the first application of the HCST to examine (i) the dietary patterns of Canadian children, and (ii) the applicability and relevance of HCST as a measure of diet quality.

**Methods:**

Data were from the nationally-representative, cross-sectional Canadian Community Health Survey 2.2. A total of 13,749 participants aged 2–18 years who had complete lifestyle and 24-hour dietary recall data were examined.

**Results:**

Dietary patterns of Canadian children and adolescents demonstrated a high prevalence of Tier 4 foods within the sub-groups of processed meats and potatoes. On average, 23–31 % of daily calories were derived from “other” foods and beverages not recommended in EWCFG. However, the majority of food choices fell within the Tier 2 and 3 classifications due to lenient criteria used by the HCST for classifying foods. Adherence to the recommendations presented in the HCST was associated with closer compliance to meeting nutrient Dietary Reference Intake recommendations, however it did not relate to reduced obesity as assessed by body mass index (*p* > 0.05).

**Conclusions:**

EWCFG recommendations are currently not being met by most children and adolescents. Future nutrient profiling systems need to incorporate both positive and negative nutrients and an overall score. In addition, a wider range of nutrient thresholds should be considered for HCST to better capture product differences, prevent categorization of most foods as Tiers 2–3 and provide incentives for product reformulation.

**Electronic supplementary material:**

The online version of this article (doi:10.1186/s12889-016-3038-5) contains supplementary material, which is available to authorized users.

## Background

Nutrition plays a critical role in growth and development of children and youth, where both under and over nutrition can have serious health implications [1]. Currently, there is strong evidence that dietary behaviours are developed early in life, and are likely to remain stable in childhood through to the adulthood years [2–4]. Establishing healthy behaviours from early childhood helps prevent obesity and several other chronic diseases [5]; and therefore, the dietary habits of children is currently one of the most pressing public health issues [1]. Even though many studies have evaluated infant feeding practices [6–8], research on diet quality shortly after the lactation and weaning period remains scarce [9].

Countries worldwide have implemented dietary guidelines and included recommendations specific to children to promote healthful dietary practices, help develop long-term positive eating behaviours and prevent obesity [10–12]. While guidelines are in place, the ability to evaluate actual dietary practices and adherence to guidelines is essential for population nutrition monitoring. According to the World Health Organization (WHO), nutrient profiling of foods, described as “the science of classifying or ranking foods according to their nutritional composition for reasons related to preventing chronic disease and promoting health” [13], is becoming the basis for regulating health claims on food packages, fortification, as well as marketing and advertising to children [14]. In 2015, the WHO developed a nutrient profiling model for Europe based on the many systems currently in use worldwide [13, 15–18]. To date, nutrient profiling systems have mainly been applied to food products and used by the food industry; yet addressing the nutritional profiles of dietary consumption is important for evaluating guidelines aimed at improving the eating patterns at the “population” level [19].

In 2014, Health Canada Surveillance Tool (HCST) Tier System [19] was developed akin to nutrient profiling to assess the food intakes of Canadians relative to Eating Well with Canada’s Food Guide (EWCFG) guidance [20] based on the classification of foods in the Canadian Nutrient File (CNF) [21]. This tool aims to assess dietary adherence to EWCFG in terms of amount and type of foods recommended (i.e. number of servings from each food group, and within these, the quality of food choices) [19]. Details regarding the HCST have been previously published by Health Canada [19]. HCST is the first government-developed nutrient profiling system in Canada, and is a categorical system that classifies foods within each food group into four Tiers according to their adherence with EWCFG recommendations [19].

Health Canada notes that HCST is for surveillance of dietary intakes [19]; however, this has yet to be applied to the eating habits of Canadian children and adolescents and its applicability and relevance is not currently established. As well, no previous study in Canada has applied nutrient profiling systems to evaluate eating habits of children and adolescents in relation to the EWCFG. Thus, the objectives of this study are two-fold; 1) to use HCST Tier system to evaluate the eating habits of Canadian children and adolescents using dietary data from the Canadian nationally-representative nutrition survey; and 2) to gauge the applicability and relevance of this tool on a population basis.

## Methods

Data for this study were unidentifiable from the Canadian Community Health Survey, Cycle 2.2 (CCHS), conducted by Statistics Canada under the authority of the Statistics Act of Canada [22, 23]. All data analyses were performed at a Statistics Canada Research Data Center. The CCHS 2.2 is a complex multi-stage, stratified, population-based survey, conducted in 2004–05, targeting respondents from all age groups across the 10 Canadian provinces (*n = 35,107*) [22]. Canada has 10 provinces (Alberta, British Columbia, Manitoba, New Brunswick, Newfoundland and Labrador, Nova Scotia, Ontario, Prince Edward Island, Quebec, and Saskatchewan) and 3 northern territories, and only provinces participated in this survey. This survey is the latest national Canadian nutrition survey since the Nutrition Canada survey, which was conducted in 1972 [22]. The sampling method was designed to be representative of the population in terms of age, sex, geography, and socioeconomic status. Residents of the three territories, people living on First Nations reserves or Crown lands, individuals living in institutions, and residents of some remote regions were excluded. Further details regarding the study design, sampling frameworks, and procedures were reported previously [22, 23]. To address the first objective of this study, data obtained from all children and adolescent respondents (2–18 years) who had valid dietary recalls (as defined by Statistics Canada) and were non-pregnant and non-lactating were included (*n = 13,749*). Additionally, for evaluation of the applicability and relevance of HCST (objective 2) and calculation of estimated energy requirement (EER) needed for identifying misreported dietary recalls, individuals with missing height, weight, and physical activity measurements were excluded *(n = 8,864)*.

### Data collection

Detailed dietary intake data were collected by means of a 24-hour recall using a 5-step modified version of the US Department of Agriculture (USDA) Automated Multiple Pass Method (AMPM) administered by trained interviewers [22, 23]. Energy and nutrient composition of reported foods were derived from Health Canada’s CNF [21], which is based on the USDA Nutrient Database for Standard Reference (Release 13) [24] with adjustments for Canadian regulations and Canadian-specific foods and recipes [21]. Children aged ≥12 years provided their own dietary recall data, whereas children aged 6–11 years provided dietary information along with their parent or caregiver [22]. Proxy data were available for children under 6 years of age, with food intakes reported by a parent or caregiver [22].

To determine dietary energy density of the foods consumed, total energy from foods (excluding all beverages) (kilocalories) was divided by the total food weight (grams) [25–27]. Using published International Glycemic Index (GI) table, the GI values of reported foods were calculated [28, 29] and assigned to each of the Bureau of Nutritional Sciences (BNS) food categories [30] using the procedures suggested by Louie et al. and Flood et al. [31, 32]. Glycemic load was calculated by multiplying the glycemic index value by the number of grams of carbohydrate then dividing by 100 [28, 29]. Several studies have shown that diets low in GI are associated with reduced risk of several chronic diseases, including diabetes [33, 34], coronary heart disease [34, 35], cancer [36], and may also be associated with obesity [37]. Hence, since GI may be an important dietary factor in chronic disease risk it was included in these analyses as an additional indicator of diet quality.

Trained interviewers measured height and weight according to the standard protocols, and body mass index (BMI) was calculated dividing subjects’ weight (kg) by height (m) squared [22]. Descriptive analyses were stratified by sex and age categories, as defined in the IOM Dietary Reference Intakes (DRI) [38]. Physical activity for children aged 6 to 11 years was determined based on reports from the child (and/or their parent or guardian) on the number of days in a typical week they were physically active for at least 60 min each day; the CCHS Cycle 2.2 did not collect physical activity data for children <6 years of age. Physical activity by respondents aged 12 to 18 was assessed via an index representing the average daily energy expended on leisure time physical activity, these totals were used to categorize individuals as inactive, moderately active, and active (metabolic equivalents (METs)).

### Application of the HCST to dietary recalls

#### Foods recommended in the EWCFG

Dietary data were assessed using HCST which employs a “Tier” system for the classification of foods in the CNF according to EWCFG [19]. The HCST assigns foods into 4 main food groups (i.e. Vegetables and Fruits, Grain Products, Milk and Alternatives, and Meat and Alternatives) and “other” foods and beverages recommended in EWCFG (e.g., water and healthy vegetable oil) [20]. The four main food groups were then categorized into 21 food subgroups, which were then classified into one of the four Tiers based on: 1) the amount of total fat, saturated fat, sugar, and sodium, and 2) adjustments according to other EWCFG guidance [19]. Threshold levels for fats, sodium and sugars per reference amount are used for the initial placement of foods into Tiers [19]. Reference amount (RA) is a specific regulated quantity of a type of food usually eaten in one sitting, and provides a uniform basis for any specific food category[19]. Foods classified as Tier 1 and Tier 2 are considered “foods in line with EWCFG guidance”, Tier 3 foods are considered “foods partially in line with EWCFG guidance”, while foods in Tier 4 are described as “foods that are not in line with EWCFG guidance” [19]. A detailed description of the food groups and subgroups within Health Canada’s Tier system has been published previously [19] and is briefly described below.

##### *Step 1:*

Foods that do not exceed the lower threshold levels of 3 g/RA of total fat, 6 g/RA of sugars, and 140 mg/RA of sodium are classified as Tier 1 foods [19]. Tier 2 foods are foods that exceed one or two lower thresholds for total fat, sugars, or sodium, without exceeding any upper thresholds [19]. Upper thresholds include: >10 g/RA total fat, >19 g/RA sugars, >360 mg/RA sodium, and >2 g/RA saturated fat [19]. For the Tier 3 classification, the Vegetables and Fruit and Grain Products are graded differently than the Milk and Alternatives and Meat and Alternatives food groups since the latter contain more intrinsic saturated fats [19]. For the Vegetables and Fruits and Grain Products, Tier 3 foods are those that exceed all 3 lower thresholds without exceeding any upper thresholds, or foods that surpass only one upper threshold for total fat, saturated fat, sugars or sodium [19]. For the Milk and Alternatives and Meat and Alternatives food groups, foods that are beyond all three lower thresholds without exceeding any upper thresholds for total fat, sugars or sodium; or exceed only one out of these 3 upper thresholds irrespective of saturated fat content, are considered Tier 3. For these two food groups, foods that only exceed the upper saturated fat threshold are also categorized as Tier 3 [19]. Tier 4 foods are foods that exceed at least two upper thresholds for total fat, sugars, sodium, or saturated fat, where similar to Tier 3 classification, the saturated fat content is disregarded for the Milk and Alternatives and Meat and Alternatives food groups [19].

##### *Step 2:*

Further adjustments are made according to other EWCFG guidance after the thresholds for fats, sugars, and sodium are applied, for instance, whole grains with naturally-occurring oils that exceed the lower threshold for fat are adjusted from Tier 2 to Tier1 [19].

#### Foods not recommended in the EWCFG

According to Health Canada, foods that are part of the 4 main food groups (i.e. Vegetables and Fruits, Grain Products, Milk and Alternatives, and Meat and Alternatives) as well as water and healthy vegetable oil are recommended in the EWCFG [19]. The remaining food items were classified as “other” foods and beverages not recommended in the EWCFG, and were subdivided as: a) high fat and/or high sugar foods (e.g., candies, syrups, cholates and sauces); b) saturated and/or trans fats and oils; c) high calorie (≥40 kcal/100 g) beverages; d) low-calorie beverages (<40 kcal/100 g); e) meal replacements and supplement (e.g., energy bar); f) alcoholic beverages; and g) uncategorized (dehydrated and condensed soups; unprepared mixes, and ingredients/seasoning).

### Definition of compliance to the HCST tier system

The HCST does not provide a total sum score for ranking individuals based on their adherence to the Tier system, and therefore, we categorized individuals into the quartiles according to the percentage of energy they consumed from Tier 4 foods and “other” foods and beverages that are not recommended in the EWCFG. Those in quartile 1 had the lowest percentage of energy from Tier 4 and “other” foods and were defined as “compliers”, while those in the interquartile ranges (quartiles 2 and 3) and the highest quartile (quartile 4) were labelled as “intermediates” and “non-compliers”, respectively. Dietary and lifestyle characteristics of “compliers”, “intermediates” and “non-compliers” were compared in order to evaluate the relevance and benefits of adhering to the HCST Tier system.

### Identification of implausible reporters

Energy misreporting is an important source of systematic error which may attenuate or reverse the association of dietary factors with health outcomes [39–41], as also demonstrated in our recent study (unpublished data [42]). Each participant in this study was categorized as an under-reporter, plausible reporter or over-reporter based on the comparison of their total estimated energy requirement (EER) and their reported energy intake (EI) [41]. The IOM factorial equations, which were used to estimate the EER, are developed based on a meta-analysis of studies using doubly-labeled water for EER measurement [38], and they require age, sex, weight, height, and physical activity level (PAL) (sedentary, low active, moderately active, highly active) for estimating the EER [38]. We applied McCrory et al.’s intervals to four different physical activity levels reported by children and adolescents (6–18 years). Based on our dataset for individuals <12 years, under-reporters were classified as individuals whose EI was <74 % of their EER and over-reporters were those whose EI was >135 % of their EER (±1 standard deviation). For respondents ≥12 years, individuals whose EI was less than 70 % of their EER were classified as under-reporters and those whose EI was more than 142 % of their EER were considered over-reporters (±1 standard deviation). Equations used for these calculations have been previously published [39, 41]. This is the first endeavor of addressing dietary recall misreporting among Canadian children.

### Statistical analyses

Statistical Analysis System (SAS) software (version 9.4; SAS Institute Inc, Cary, NC) was used to perform all statistical analyses. All analyses were weighted to obtain estimates at a population level. Survey weights were calculated by Statistics Canada based on respondent classes with similar socio-demographic characteristics to maintain a nationally representative sample. The bootstrap balanced repeated replication (BRR) technique was used, as recommended by Statistics Canada, to account for the complex survey design [43, 44]. To assess the lifestyle and dietary characteristic of participants, PROC SURVEYREG and PROC SURVEYLOGISTIC were used for analyzing continuous and categorical variables, respectively. Group comparison with Tukey post-hoc adjustment was used to evaluate the characteristics of participants classified within DRI age and sex categories.

Quartile analysis was conducted where individuals were stratified based on percentage of energy from Tier 4 and “other” food intake (i.e. foods that are not classified into one of the EWCFG four food groups) as defined by EWCFG [19]. Covariates included in the analysis were age, sex, and dietary recall misreporting status (i.e. under-reporter, plausible reporter, or over-reporter). Results with a two-tailed p-value <0.05 were considered statistically significant.

## Results

### Quantity of foods consumed

The number of servings consumed from Tiers 1–3 in each of the four food groups did not meet EWCFG recommendations, especially for the Vegetables and Fruits group, and is most noticeable in the older age groups (Table 1). For comparison purposes, servings contributed by Tiers 1–3 and Tiers 1–4 were assessed considering that Tier 4 foods are not technically considered as providing a serving towards EWCFG food group by Health Canada. Within the Grain Products, Milk and Alternatives, and Meat and Alternatives food groups, children aged 2 to 8 years consumed more than the recommended number of servings from Tiers 1–3. Recommended servings of Meat and Alternatives were met by both boys and girls aged 9 to 13 years, while the recommended EWCFG servings from the other three food groups were not met based on Tier 1–3 servings. As illustrated in Table 2, in general boys 14–18 years of age and girls 9–13 years consumed the highest amount of energy from Tier 4 foods at 356 kcal/day and 262 kcal/day, respectively. High fat and/or sugar foods and high-calorie beverages were the major contributors to the energy from “other” foods not recommended in the EWCFG. In total, 23–31 % (363–940 kcal) of total calorie intakes among 2–18 year old Canadians were derived from Tier 4 foods and “other” foods not recommended in the EWCFG.

**Table 1:** Weighted analysis of number of servings from Health Canada’s Eating Well with Canada’s Food Guide (EWCFG) [20] presented based on the 2014 Health Canada’s Surveillance Tool (HCST) Tier system [19] among Canadians <19 years^a,b^

	2–3 years (boys and girls)		4–8 years (boys and girls)		9–13 years (boys)		9–13 years (girls)		14–18 years (boys)		14–18 years (girls)	
Number of servings/day	Mean	SEM	Mean	SEM	Mean	SEM	Mean	SEM	Mean	SEM	Mean	SEM
Vegetables and Fruits												
Tiers 1–3	4.95	0.16^c^	4.53	0.17^c^	4.10	0.21^d^	4.59	0.22^d^	3.94	0.23^e^	4.58	0.20^e^
Tiers 1–4	5.16	0.16^c^	4.76	0.17^c^	4.43	0.21^d^	4.87	0.22^d^	4.33	0.23^e^	4.91	0.20^e^
EWCFG Recommendation	4		5		6		6		8		7	
Grain Products												
Tiers 1–3	4.85	0.14^c^	5.56	0.1^c^	5.69	0.20	5.50	0.17^d^	5.73	0.21^e^	5.43	0.17^e^
Tiers 1–4	5.90	0.15^c^	6.80	0.17^c^	6.82	0.22	6.76	0.18^d^	6.60	0.24^e^	6.53	0.19^e^
EWCFG Recommendation	3		4		6		6		7		6	
Milk and Alternatives												
Tiers 1–3	3.09	0.09^c^	2.64	0.09^c^	2.35	0.11	2.26	0.10	1.86	0.13	1.94	0.10
Tiers 1–4	3.30	0.09^c^	2.83	0.09^c^	2.55	0.12	2.46	0.10	2.10	0.13	2.15	0.10
EWCFG Recommendation	2		2		3–4		3–4		3–4		3–4	
Meat and Alternatives												
Tiers 1–3	1.28	0.08	1.34	0.09	1.57	0.12	1.39	0.10^d^	1.89	0.12^e^	1.52	0.10^e^
Tiers 1–4	1.61	0.07	1.68	0.08	1.92	0.11	1.71	0.09^d^	2.20	0.11^e^	1.76	0.10^e^
EWCFG Recommendation	1		1		1–2		1–2		3		2	

*Abbreviations: EWCFG* Eating Well with Canada’s Food Guide, *RA* Reference Amount, *SEM* Standard Error of Mean

^a^Energy adjusted

^b^Tiers are based on Health Canada’s Surveillance Tool [19] and defined generally as follows: Tier 1–3 foods are compliant with EWCFG and Tier 4 foods are not recommended by the EWCFG. Tier 1 are foods that do not exceed lower thresholds for total fat, sugars, and sodium; Tier 2 foods do not exceed up to 2 lower thresholds for total fat, sugars or sodium, without exceeding any upper thresholds; for the Vegetables and Fruit and Grain Products food groups Tier 3 are foods that exceed all 3 lower thresholds without exceeding any upper thresholds or exceed only one upper threshold, while Tier 4 foods exceed at least 2 upper thresholds for total fat, saturated fat, sugars, or sodium. Within the Milk and Alternatives and Meat and Alternatives food groups, Tier 3 foods exceed all 3 lower thresholds without exceeding any upper thresholds for total fat, sugars, or sodium (irrespective of saturated fat) or exceed only one of these 3 thresholds or foods that only exceed the upper saturated fat threshold; within these 2 food groups foods that exceed at least 2 upper thresholds for total fat, sugars, or sodium were classified as Tier 4. Where lower thresholds entail: total fat≤3 g/RA, sugars ≤6 g/RA, and sodium ≤140 mg/RA; and upper thresholds are: total fat >10 g/RA, sugars >19 g/RA, sodium >360 mg/RA, and saturated fat >2 g/RA

^c^Comparison significantly different between 2–3 year old and 4–8 year olds, based on Tukey multiple comparison test (*p* < 0.05)

^d^Comparison significantly different between 9–13 year old males and females, based on Tukey multiple comparison test (*p* < 0.05)

^e^Comparison significantly different between 14–18 year old males and females, based on Tukey multiple comparison test (*p* < 0.05)

**Table 2:** Weighted analysis of energy contribution from Tiers 1–3 foods (compliant with Eating Well with Canada’s Food Guide (EWCFG)) [19] and Tier 4 and “other” foods and beverages [19] not included in the EWCFG among Canadians (<19 years)^a^

	2–3 years (boys and girls)		4–8 years (boys and girls)		9–13 years (boys)		9–13 years (girls)		14–18 years (boys)		14–18 years (girls)	
Variable (kcal/day)	Mean	SEM	Mean	SEM	Mean	SEM	Mean	SEM	Mean	SEM	Mean	SEM
Tiers 1 + 2 + 3^b^	999	16	1142	14	1395	24	1160	19	1621	26	1144	19
Tier 4^c^	165	8	239	7	33	14	262	12	356	14	240	11
Other Foods												
Alcoholic beverages^d^	0	0	1	0	0	0	0	0	36	11	15	2
Beverages, higher calorie (≥40 kcal/100 g)^e^	60	4	90	4	149	6	126	5	227	9	144	7
Beverages, lower calorie (<40 kcal/100 g)^f^	7	1	12	1	22	2	18	2	33	3	25	3
High fat and/or sugar foods^g^	93	10	122	4	195	11	165	9	191	10	168	12
Meal replacements^h^	1	0	1	1	2	1	1	0	7	3	2	1
Saturated and/or trans fats and oils^i^	31	2	44	2	60	4	47	3	78	5	52	3
Supplements^j^	1	1	1	1	0	0	0	0	1	0	1	0
Uncategorized ingredients, seasonings and unprepared foods^k^	7	1	8	1	13	1	14	2	19	2	15	1
Unsaturated fats and oils^l^	23	2	33	2	51	4	39	3	72	4	61	4
Total energy from Tier 4 and “other” foods (kcal/day)^m^	363	14	515	10	770	20	632	17	940	27	659	19
Total (% of total Tier 4 and “other” foods)^n^	23	1	27	0	31	1	30	1	31	1	31	1

*Abbreviations: EWCFG* Eating Well with Canada’s Food Guide, *SEM* Standard Error of Mean

^a^“Other foods” are not part of the Tier system and include “other” food and beverages in the Eating Well with Canada’s Food Guide main food groups, meal replacements, and supplements

^b^All age and sex comparisons were significant, except for: the difference between 4–8 and 9–13 year old boys; 4–8 year olds and 14–18 year old girls; and 9–13 year old and 14–18 year old girls

^c^All age and sex comparisons were significant, except for: the difference between 4–8 and 9–13 year old girls; 4–8 year olds and 14–18 year old girls; 9–13 year old and 14–18 year old boys; and 9–13 year old and 14–18 year old girls

^d^All age and sex comparisons were significant, except for: the difference between 2–3 year olds and 4–8 year olds; 2–3 year olds and 9–13 year old boys; 2–3 year olds and 9–13 year old girls; 4–8 year olds and 9–13 year old boys; 4–8 year olds and 9–13 year old girls; 9–13 year old boys and 9–13 year old girls

^e^All age and sex comparisons were significant, except for: the difference between 9–13 year old boys and 14–18 year old girls

^f^All age and sex comparisons were significant, except for: the difference between 9–13 year old boys and 9–13 year old girls; 9–13 year old boys and 14–18 year old girls; 9–13 year old girls and 14–18 year old girls; 14–18 year old boys and 14–18 year old boys

^g^All age and sex comparisons were significant, except for: the difference between 9–13 year old boys and 14–18 year old boys; 9–13 year old boys and 14–18 year old girls; 9–13 year old girls and 14–18 year old boys; 9–13 year old girls and 14–18 year old girls; 14–18 year old boys and 14–18 year old boys

^h^All age and sex comparisons were not significant, except for: the difference between 2–3 year olds and 9–13 year old boys; 2–3 year olds and 14–18 year old boys

^i^All age and sex comparisons were significant, except for: the difference between 4–8 year olds and 9–13 year old girls; 4–8 year olds and 14–18 year old girls; 9–13 year old boys and 14–18 year old girls; 9–13 year old girls and 14–18 year old girls

^j^All age and sex comparisons were not significant, except for: the difference between 4–8 year olds and 9–13 year old girls; 9–13 year old girls and 14–18 year old girls

^k^All age and sex comparisons were significant, except for: the difference between 2–3 year olds and 4–8 year olds; 9–13 year old boys and 9–13 year old girls; 9–13 year old boys and 14–18 year old girls; 9–13 year old girls and 14–18 year old boys; 9–13 year old girls and 14–18 year old girls; 14–18 year old boys and 14–18 year old girls

^l^All age and sex comparisons were significant, except for: the difference between 4–8 year olds and 9–13 year old girls; 9–13 year old boys and 14–18 year old girls; 14–18 year old boys and 14–18 year old girls

^m^All age and sex comparisons were significant, except for: the difference between 9–13 year old girls and 14–18 year old girls

^n^All age and sex comparisons were significant, except for: the difference between 9–13 year old boys and 9–13 year old girls; 9–13 year old boys and 14–18 year old boys; 9–13 year old boys and 14–18 year old girls; 9–13 year old girls and 14–18 year old boys; 9–13 year old girls and 14–18 year old girls; 14–18 year old boys and 14–18 year old girls

### Quality of food consumed

**Fig. 1:**
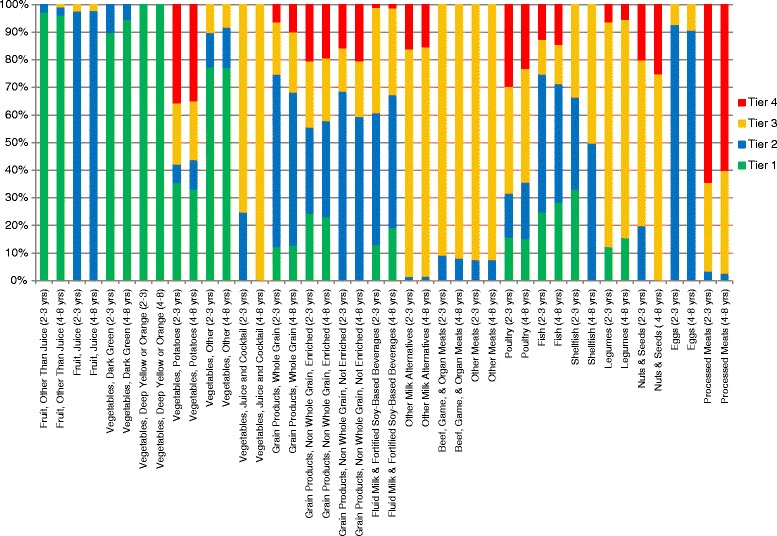
Weighted age-stratified analysis of classification of foods as a percentage of servings based on the 2014 Health Canada Surveillance Tool Tier system^a,b^ among Canadian population of Boys and Girls ages 2 to 8 years. ^a^Energy adjusted ^b^Tiers are based on Health Canada’s Surveillance Tool [19] and defined generally as follows: Tier 1–3 foods are compliant with EWCFG and Tier 4 foods are not recommended by the EWCFG. Tier 1 are foods that do not exceed lower thresholds for total fat, sugars, and sodium; Tier 2 foods do not exceed up to 2 lower thresholds for total fat, sugars or sodium, without exceeding any upper thresholds; for the Vegetables and Fruit and Grain Products food groups Tier 3 are foods that exceed all 3 lower thresholds without exceeding any upper thresholds or exceed only one upper threshold, while Tier 4 foods exceed at least 2 upper thresholds for total fat, saturated fat, sugars, or sodium. Within the Milk and Alternatives and Meat and Alternatives food groups, Tier 3 foods exceed all 3 lower thresholds without exceeding any upper thresholds for total fat, sugars, or sodium (irrespective of saturated fat) or exceed only one of these 3 thresholds or foods that only exceed the upper saturated fat threshold; within these 2 food groups foods that exceed at least 2 upper thresholds for total fat, sugars, or sodium were classified as Tier 4. Where lower thresholds entail: total fat ≤3 g/RA, sugars ≤6 g/RA, and sodium ≤140 mg/RA; and upper thresholds are: total fat >10 g/RA, sugars >19 g/RA, sodium >360 mg/RA, and saturated fat >2 g/RA

The majority of Vegetables and Fruits consumed, except from the potato subgroup, were classified as Tier 1 among all age and sex groups (Fig. 1 and Additional file 1: Figure S1). Conversely, the processed meat and potato subgroups contained the highest proportions of Tier 4 food choices. Using the HCST Tier system, the majority of the remaining Grain Products, Milk and Alternatives, and Meat and Alternatives subgroup foods were categorized as Tiers 2 and 3. In general, the pattern of food consumption was consistent within different age groups, even though food choices ranged from “healthy” (Tier 1) to poor food choices (Tier 4). Additional file 1: Table S1 illustrates the percentage of energy intake within the Fruits and Vegetables, Grains Products, Milk and Alternatives, and Meat and Alternatives food groups according to the HCST in children and adolescents, respectively. In children (2–11 years), Tier 1 whole fruits and Tier 2 fruit juices were the main sources of calorie intake providing 50 and 46 % of Fruit sub-group calories, respectively. Within the Vegetable sub-group, the majority of calories were from Tier 4 (27 %) and Tier 3 (21 %) potatoes, followed by Tier 1 other vegetables (18 %). When united into the Vegetable and Fruit Group, as per the EWCFG system, over 50 % of calories came from Tier 1 fruit other than juice (28 %), Tier 2 fruit juice (25 %), and Tier 4 potatoes (12 %). Seventy-four percent of calories from the Grain Products group was contributed by enriched, non-whole grains, compared to 13 % coming from whole grains. Fluid milk and fortified soy-based beverages from Tiers 2 and 3 were the major calorie contributors within the Milk and Alternatives group. Within the Meat group (i.e. excluding Meat Alternatives), Tier 3 beef, game and organ meat provided 26 % of calories, while Tier 4 processed Meats contributed 23 %. When considering Meat Alternatives alone, Tier 3 legumes (33 %) and Tier 2 eggs (26 %) were the main energy sources. However, when united into the Meats and Alternatives food group, percentage of calories contributed by the alternatives became relatively less with the top providers being Tier 3 beef, game and organ meats (19 %), Tier 4 processed meats (17 %), and Tier 3 poultry (10 %).

Adolescents (12–18 years) presented a similar eating pattern to younger children (2–11 years) with regards to percentage of calories from the EWCFG food groups (Additional file 1: Table S1). Within the Vegetables and Fruits food group, while Tier 1 fruit other than juice, and Tier 1 other vegetables contributed 30 % of food group calories, over 50 % of energy were derived by non-Tier 1 foods, specifically Tier 2 fruit juice (26 %), Tier 4 potatoes (16 %), and Tier 3 potatoes (11 %). Non-whole, enriched grains made up 75 % of Grain Products calories, with Tiers 2 and 4 being the most prominent at 27 % and 18 %, respectively. Fluid Milk and fortified soy-based beverages (Tier 2) were the main sources of calories from the Milk and Alternatives food group (45 %). Analogous to children, Tier 3 beef, game and organ meats (30 %) and Tier 4 processed meats (18 %) were the top calorie contributors when assessing the Meat subgroup solely; while, within the Meat Alternatives compilation, Tier 3 legumes (35 %) and Tier 2 eggs (24 %) were the main suppliers of calories. As well, when Meat and Alternatives were assembled into one food group, as determined by EWCFG recommendations, Tier 3 beef, game and organ meats (23 %), Tier 4 processed meats (14 %), and Tier 3 poultry (11 %) were the main energy suppliers.

### Association between percentage of energy from tier 4 and “other” foods and obesity

For children (2–11 years), as indicated in Table 3, individuals with the lowest percentage energy from Tier 4 foods and “other” foods (quartile 1; compliers) were more likely to be younger compared to those in the fourth quartile (non-compliers) (p-trend <.0001). In adolescents (12–18 years), individuals in quartile 1 (compliers) tended to be non-smokers compared to those in the fourth quartile (non-compliers) (Additional file 1: Table S2) (p-trend <.0019). However, there was no significant trend between BMI measures and quartiles of percentage energy from Tier 4 and “other” foods among children and adolescents (p-trend >0.78). Additional age- and sex- (with and without misreporting) adjusted regression analysis did not reveal any significant associations (odds ratio for quartile 4 vs. quartile 1: 0.72 (0.417–1.245) in children (p-trend = 0.229); 0.969 (0.602–1.561) in adolescents (p-trend = 0.6605) (data not shown).

**Table 3:** Weighted analysis of characteristics of compliers, intermediates, and non-compliers based on the percentage of energy from Tier 4 foods and “other” foods among Canadian children (≥2 to <12 years)^a, b^

	Compliers (Q1)^c^		Intermediates (Q2)^d^		Intermediates (Q3)^d^		Non-compliers (Q4)^e^		
	≤22.29 % Energy		22-29-33.83 % Energy		33.83-47.48 % Energy		>47.48 % Energy		
Characteristics	Mean	SEM	Mean	SEM	Mean	SEM	Mean	SEM	P-Trend
Age (years)	6.55	0.23	7.09	0.28	7.31	0.30	7.61	0.29	<.0001
Sex									
Males (%)	49.60	2.81	53.55	3.16	52.21	2.55	48.12	2.57	
Females (%)	50.40	2.81	46.45	3.16	47.79	2.55	51.88	2.57	0.3034
BMI (kg/m^2^)	17.64	0.17	17.78	0.16	17.55	0.15	17.41	0.12	0.3100
Misreporting Status									
Under Reporters (%)	11.72	1.88	9.89	1.65	5.80	1.00	6.48	1.13	
Plausible Reporters (%)	56.60	2.73	52.64	2.79	54.19	2.51	49.79	2.94	
Over Reporters (%)	31.69	2.45	37.47	2.62	40.01	2.47	43.73	3.04	0.0015
Physical Activity (%)^f^									
(60 min, 0 days/wk)	3.18	1.46	0.76	0.43	1.75	0.84	1.75	0.93	
(60 min, 1 day/wk)	1.60	0.87	2.05	1.26	2.75	0.98	2.88	1.06	
(60 min, 2–3 days/wk)	18.09	3.17	15.31	3.16	17.03	2.67	13.42	2.30	
(60 min, 4+ days/wk)	77.13	3.37	81.88	3.23	78.47	2.81	81.95	2.54	0.3849

*Abbreviation: SEM* Standard Error of Mean

^a^Adjusted for age and sex

^b^Quartiles are based upon percentage of energy from all Tier 4 foods based on Health Canada’s Surveillance Tool Tier system 2014 plus “other” foods and beverages not recommended in the Eating Well with Canada’s Food Guide

^c^The 25 % of individuals with the lowest percentage of energy from Tier 4 and “other” foods

^d^The individuals in the interquartile range for energy intakes from Tier 4 and “other” foods

^e^The 25 % of individuals with the highest percentage of energy from Tier 4 and “other” foods

^f^Physical activity level is assessed for individuals >6 yrs

### Association between the percentage of energy from tier 4 foods and “other” foods and overall dietary nutrient density

**Fig. 2:**
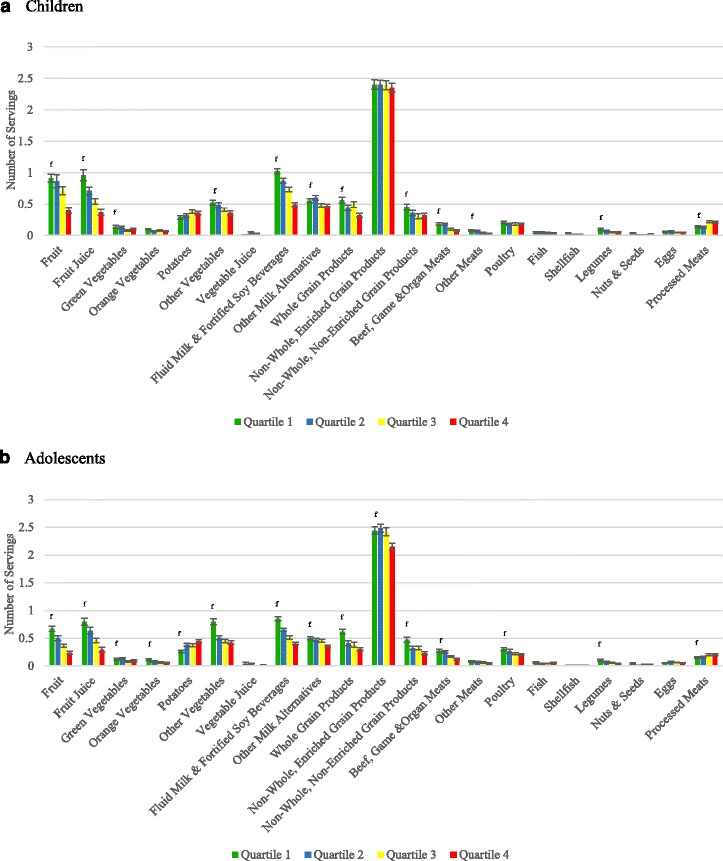
Implementation of Health Canada Surveillance Tool Tier system applied to the dietary intakes of Canadians <19 years in a weighted analysis of Children (≥2 to <12 years) and Adolescents (≥12 to <19 years). Dietary profile of compliers (Quartile 1)^a^, intermediates (Quartiles 2 and 3)^b^, and non-compliers (Quartile 4)^c^ based on the serving from each of the Eating Well with Canada’s Food Guide subgroups per 1000 Kcal for **a**) Children (2–11 years), **b**) Adolescents (12–18 years)^d,e^ ^a^The 25 % of individuals with the lowest number of Tier 4 and “other” food servings ^b^The individuals in the interquartile range for dietary intake of Tier 4 and “other” food servings ^c^The 25 % of individuals with the highest number of Tier 4 and “other” food servings ^d^Adjusted for age, sex, and misreporting status (under-reporter, plausible-, and over-reporters) ^e^Quartiles are based upon percentage of energy from all Tier 4 foods based on Health Canada’s Surveillance Tool Tier system 2014 plus “other” foods and beverages not recommended in the Eating Well with Canada’s Food Guide ^f^Significant p-value for trend for difference of quartiles (P-trend <0.05)

The total servings from EWCFG food subgroups per 1000 kcal is presented in a graph among compliers (Q1), intermediate compliers (both Q2 and Q3), and non-compliers (Q4) (Fig. 2). In the bar graph each of the 4 quartiles (based on the percentage of energy from Tier 4 foods and other foods not recommended in the EWCFG) are presented for each of the food sub-groups. After adjusting for age, sex and misreporting status, in most cases compliers (Q1; represented by green column) had higher servings of Fruits, Vegetable subgroups (including subgroups), Milk and Alternatives, Grains Products, and Meat and Alternatives per 1000 kcal compared to the non-complier group (Q4, represented by the red column). Adolescents who consumed the highest percentage of energy from Tier 4 foods and “other” foods (non-compliers) had the highest intakes of potatoes (0.44 ± 0.0321) compared to those in the lowest quartile (compliers) (0.26 ± 0.02269) (p-trend <.0001); this was similar to children even though the trend did not reach statistical significance (p-trend = 0.1046). In contrast, compliers had higher servings of Fruits, Vegetables (including subgroups), Milk and Alternatives, Grains Products, and Meat and Alternatives per 1000 kcal compared to the non-complier group, even though some trends were not significant  despite having consistent trends.

In children (Table 4), compliers consumed significantly less calories (252 kcal/day on average) compared to non-compliers, which may be related to the trend of lower energy consumption from fat, saturated fat, mono-unsaturated fatty acid, poly-unsaturated fatty acid, and added sugars in the complier (Q1) compared to the non-complier (Q4) group (*P* < 0.05). It is noteworthy however, that even though a 252 kcal difference in calories between compliers and non-compliers is likely clinically-relevant for weight maintenance/loss over time, a 1 % difference in percentage of saturated fat may not be clinically-relevant. Dietary fiber (Q1: 16.13 % vs. Q4:11.55 %) and protein intake (Q1:8.30 % vs. Q4:6.21 %) were also higher in compliers compared to non-compliers (*P* < 0.0001). In terms of micronutrients (food sources only), the intake of vitamin A, vitamin D, vitamin B1, vitamin B6, vitamin B12, folate, and vitamin C were significantly higher in the compliers (Q1) than the non-compliers (Q4) (P <0.01). Similarly, mineral intakes including calcium, phosphorus, potassium, magnesium, iron, and zinc, were significantly higher in the compliers compared to the non-compliers (*P* < 0.0001); whereas, quartile 3 (intermediate compliers) had the highest sodium intake (*P* < 0.01). In addition, glycemic index, glycemic load, and energy density were lower in the complier group as compared to the non-compliers (*P* < 0.0001).

**Table 4:** Weighted analysis of nutrient intakes (density approach) [56] by compliers, intermediates, and non-compliers based on the percentage of energy consumed from Tier 4 foods and “other” foods among Canadian children (2 to 11 years)^a,^ ^b^

		Compliers (Q1)^e^		Intermediates (Q2)^f^		Intermediates (Q3)^f^		Non-compliers (Q4)^g^		
		≤22.29 % Energy		22-29-33.83 % Energy		33.83-47.48 % Energy		>47.48 % Energy		
Nutrients		Mean	SEM	Mean	SEM	Mean	SEM	Mean	SEM	P-Trend
Energy (kcal/day)	c	1863	42	1996	36	2026	30	2116	49	0.00
	d	1732.13	24.75	1799.65	21.80	1774.36	23.62	1834.42	27.44	0.05
Fat (%Energy)	c	28.14	0.38	30.39	0.36	30.93	0.35	33.07	0.44	<.0001
	d	27.59	0.37	29.67	0.38	30.04	0.36	32.13	0.44	<.0001
Saturated fat (%Energy)	c	10.55	0.19	11.08	0.21	11.10	0.16	11.68	0.21	0.00
	d	10.35	0.20	10.83	0.22	10.79	0.19	11.35	0.22	0.00
Monounsaturated fat (%Energy)	c	10.28	0.18)	11.56	0.17	11.97	0.18	12.95	0.21	<.0001
	d	10.06	0.17	11.27	0.19	11.61	0.18	12.57	0.21	<.0001
Polyunsaturated fat (%Energy)	c	4.35	0.13	4.64	0.08	4.94	0.09	5.37	0.15	<.0001
	d	4.26	0.13	4.52	0.10	4.80	0.09	5.23	0.14	<.0001
Carbohydrates (%Energy)	c	55.58	0.49	54.46	0.40	55.71	0.44	55.19	0.51	0.15
	d	56.28	0.49	55.33	0.43	54.78	0.46	56.31	0.52	0.08
Added sugar (%Energy)	c	10.81	0.38	12.24	0.42	14.79	0.50	17.20	0.59	<.0001
	d	10.84	0.43	12.28	0.47	14.84	0.58	17.26	0.66	<.0001
Dietary fiber (g/1000 kcal)	c	8.19	0.17	7.36	0.15	6.90	0.15	6.01	0.12	<.0001
	d	8.30	0.19	7.50	0.18	7.08	0.18	6.21	0.16	<.0001
Protein (%Energy)	c	16.28	0.21	15.09	0.21	13.35	0.17	11.71	0.16	<.0001
	d	16.13	0.22	14.94	0.21	13.17	0.19	11.55	0.17	<.0001
Alcohol (%Energy)	c	0.00	0.00	0.06	0.04	0.01	0.00	0.02	0.01	0.11
	d	0.00	0.00	0.06	0.04	0.01	0.00	0.02	0.01	0.14
Vitamin A (RE/1000 kcal)	c	348.98	9.25	334.97	10.17	296.11	7.47	259.22	7.56	<.0001
	d	348.61	9.67	335.92	11.47	297.65	8.40	261.61	8.15	<.0001
Vitamin D (ug/1000 kcal)	c	3.62	0.11	3.36	0.11	2.92	0.09	2.45	0.08	<.0001
	d	3.67	0.14	3.42	0.12	3.00	0.11	2.53	0.10	<.0001
Thiamin (mg/1000 kcal)	c	0.99	0.02	0.89	0.02	0.79	0.01	0.68	0.01	<.0001
	d	1.00	0.02	0.90	0.02	0.80	0.02	0.69	0.02	<.0001
Riboflavin (mg/1000 kcal)	c	1.15	0.01	1.08	0.02	1.00	0.02	0.93	0.03	<.0001
	d	1.16	0.02	1.10	0.02	1.03	0.02	0.95	0.04	<.0001
Niacin (NE/1000 kcal)	c	17.77	0.27	16.52	0.21	15.05	0.18	13.24	0.17	<.0001
	d	17.64	0.29	16.40	0.21	14.90	0.21	13.10	0.19	<.0001
Vitamin B6 (ug/1000 kcal)	c	0.94	0.02	0.83	0.02	0.73	0.01	0.61	0.01	<.0001
	d	0.94	0.02	0.83	0.02	0.73	0.01	0.62	0.01	<.0001
Folate (ug/1000 kcal)	c	108.16	2.86	96.56	2.56	84.59	2.21	74.28	2.19	<.0001
	d	109.22	3.31	98.12	2.84	86.58	2.60	76.48	2.80	<.0001
Vitamin B12 (ug/1000 kcal)	c	2.17	0.09	2.10	0.10	1.86	0.11	1.44	0.04	<.0001
	d	2.13	0.09	2.06	0.10	1.82	0.11	1.40	0.05	<.0001
Vitamin C (mg/1000 kcal)	c	86.23	3.76	76.90	2.98	71.93	2.39	65.23	2.73	<.0001
	d	86.96	4.56	78.32	3.49	73.81	3.04	67.48	3.37	0.00
Calcium (mg/1000 kcal)	c	610.13	12.76	571.64	10.27	505.19	10.13	427.65	8.15	<.0001
	d	614.92	13.74	578.60	11.48	514.03	11.73	437.46	10.04	<.0001
Phosphorous (mg/1000 kcal)	c	740.25	9.42	693.36	8.84	620.76	8.43	543.90	7.80	<.0001
	d	742.69	10.05	697.08	9.68	625.53	9.72	549.29	9.00	<.0001
Potassium (mg/1000 kcal)	c	1580.91	24.20	1438.47	20.67	1299.15	18.56	1099.98	19.08	<.0001
	d	1592.29	24.48	1455.85	22.21	1321.45	20.19	1125.10	20.13	<.0001
Sodium (mg/1000 kcal)	c	1446.79	38.66	1438.80	30.64	1477.03	27.64	1358.75	19.94	0.00
	d	1440.79	38.37	1433.99	33.88	1471.81	30.13	1354.73	23.75	0.00
Magnesium (mg/1000 kcal)	c	156.20	2.02	139.59	1.88	127.66	1.47	110.43	1.51	<.0001
	d	157.58	2.20	141.52	2.07	130.10	1.86	113.10	1.76	<.0001
Iron (mg/1000 kcal)	c	7.47	0.13	6.95	0.11	6.48	0.10	5.78	0.08	<.0001
	d	7.53	0.14	7.02	0.12	6.56	0.11	5.86	0.10	<.0001
Zinc (mg/1000 kcal)	c	5.52	0.09	5.38	0.17	4.87	0.25	4.02	0.07	<.0001
	d	5.47	0.09	5.31	0.17	4.78	0.25	3.92	0.08	<.0001
Glycemic Index	c	52.42	0.32	53.96	0.33	56.14	0.31	57.85	0.31	<.0001
	d	52.38	0.32	53.91	0.34	56.07	0.33	57.77	0.34	<.0001
Glycemic Load	c	135.93	2.93	148.16	2.82	160.79	3.27	171.85	3.94	<.0001
	d	126.67	2.02	134.26	1.99	143.01	2.75	151.92	2.71	<.0001
Energy Density (kcal/g)	c	1.80	0.03	1.92	0.03	2.05	0.03	2.33	0.04	<.0001
	d	1.79	0.04	1.90	0.03	2.03	0.03	2.30	0.04	<.0001

*Abbreviation: SEM* Standard Error of Mean

^a^Quartiles are based upon percentage of energy from all Tier 4 foods based on Health Canada’s Surveillance Tool Tier system 2014 plus “other” foods and beverages not recommended in the Eating Well with Canada’s Food Guide

^b^Dietary data are from food sources of nutrients only

^c^Means are adjusted for age and sex

^d^Means are adjusted for age, sex, and misreporting status (under-reporter, plausible-, and over-reporters)

^e^The 25 % of individuals with the lowest percentage of energy from Tier 4 and “other” foods

^f^The individuals in the interquartile range for energy intakes from Tier 4 and “other” foods

^g^The 25 % of individuals with the highest percentage of energy from Tier 4 and “other” foods

Adolescents presented with similar trends (Additional file 1: Table S3) with compliers consuming significantly less calories (on average 429 kcal less/ day) compared to non-compliers (*p* < 0.0001).

## Discussion

### Main findings

Based on a nationally representative sample of Canadians, this study presents the first evaluation of the eating habits of Canadian children and adolescents using the HCST Tier system. These analyses are of high importance since a key application of nutritional profiling systems is to assess and guide the marketing of foods towards children [13], yet to date the effectiveness of Health Canada’s Tier system has not been critically assessed among this population. Assessment of dietary intakes via this nutrient profiling system revealed that not only are children and adolescents not meeting Health Canada’s recommended number of food group servings, their consumption of unhealthy Tier 4 classified foods are high, especially for processed meats and potatoes. In addition, about one-third on average of daily kilocalories were consumed from Tier 4 and “other” food sources not recommended in the EWCFG, even though some groups had lower intakes.

With regards to determining the applicability and relevance of the Tier system, the majority of food choices fell within the Tier 2 and 3 categories, suggesting the lack of differentiating ability of the thresholds used by the HCST Tier system. This may also explain the lack of significance observed between adherence to Health Canada’s Tier system and obesity (as measured by BMI), even though compliance to the HCST was associated with increased probability of meeting DRI nutrient recommendations. These results were not unforeseeable since the HCST Tier system was established to adhere to EWCFG [20], which itself is modeled based on achieving DRI nutrient recommendations [45, 46]. We recently showed that following the EWCFG guidance may result in energy overconsumption and eventually higher risk of overweight and obesity [45]. Thus, while closer compliance to the Tier system does reflect higher accordance to attaining the DRI reference intakes, it does not address recommendations for the prevention of chronic diseases [45]. Nevertheless, our results corroborate with those of the others, which have found neutral [47] or even inverse [48] associations between a priori diet quality indexes and risk of obesity, which may be attributed to the cross-sectional design of these studies or the fact that obese individuals are more likely to be changing their eating habits [49, 50].

### Comparisons with other nutrient profiling systems

In 2013, the WHO European Member States expressed concern regarding the high burden of chronic diseases caused by unhealthy diets, particularly the rise of overweight and obesity among children. Hence, the Vienna Declaration on Nutrition and Noncommunicable Diseases in the Context of Health 2020 was devised to take “decisive action to reduce food marketing pressure to children with regard to foods high in energy, saturated fats, trans fatty acids, free sugars or salt” and to develop and implement common policy approaches that promote, among other things, the use of common nutrient profiling tools [51]. In 2015, WHO unveiled its nutrient profiling tool intended to help national authorities identify unhealthy foods by their saturated fat, trans fat, sodium, and added sugar content and to restrict their marketing to children [15]. Compared to Health Canada’s Tier system, which contains 9 main food categories, the WHO model encompasses 17 food categories, with both systems utilizing pre-defined thresholds to classify foods [15, 19]. The HCST Tier system food categories contain multiple sub-groups, and wide-ranging, distinct definitions for defining the thresholds for Tiers 1 and 4, and lenient criteria used for the Tier 2 and 3 categorization of foods due to the adjustable tolerance of the system [19], which may be a potential weakness of this model. A consequence of this limited threshold range and adjustable criteria is a small percentage of products being appointed to the Tier 1 and 4 categories, especially in food sub-groups such as the Milk and Alternatives.

A number of existing models were considered for use and adaptation in creating the WHO nutrient profiling system, including those developed by governments in the United Kingdom, Australia and New Zealand, and the United States, some of which have been incorporated into legislation [12, 15, 17, 52, 53]. The Ofcom nutrient profiling system of the United Kingdom determines a total dietary score based on a calculation incorporating both “negative” nutrient sources (energy, total sugar, saturated fat, sodium) and “positive” nutrient sources (fruits, vegetables and nuts, fiber, and protein) [17]. Likewise, the Nutrient Profiling Scoring Criterion (NPSC), developed by Food Standards Australia New Zealand (FSANZ), regulates health claims in Australia and New Zealand [12], and in comparison to Health Canada’s Tier system, not only does the NPSC take into account the saturated fat, sodium and sugar content of food, but it also accounts for energy content along with certain components such as fruit and vegetables, and in some instances, dietary fiber and protein leading to the calculation of an overall nutrient profiling score for a food [12]. The United States takes a similar approach with their NuVal Nutritional Scoring System based on the Overall Nutritional Quality Index (ONQI) algorithm [52, 53]. The ONQI summarizes comprehensive nutritional information into a single score of relative nutrition and healthfulness ranging from 1 to 100 based on over 30 nutrients and food properties, with evidence suggesting that a higher ONQI dietary score is associated with a moderately lower risk of chronic diseases and overall mortality [54]. In contrast to these systems, the Canadian HCST Tier system focuses on only four “negative” nutrients (total fats, saturated fat, sugars, and sodium) and lacks a total dietary score calculation for comparison purposes.

### Strengths and limitations

A major strength of this study is that it represents the most up to date and comprehensive analysis of the dietary patterns of Canadian children and adolescents using the Health Canada Surveillance Tool (HCST) and a large nationally-representative sample, including several covariates, measured anthropometry, and the use of the USDA Automated Multi-Pass Method which minimized misreporting bias.

Weaknesses of this study include the fact that the CCHS data was collected in 2004/2005 (despite being the latest Canadian survey) in addition to the limitation of the day-to day variation (random non-differential error) associated with 24-hour dietary recalls. Also, different assessment questions were used to measure physical activity level in the children and adolescent groups [22]. Physical activity could not be assessed in children less than 6 years of age because this data was not collected in the CCHS, Cycle 2.2 survey. Another limitation common among diet quality index analyses was the subjectivity surrounding the selection of nutritional components, scoring criteria, and threshold values [55].

### Implications for future Canadian nutrient profiling models

Based on the present evaluation of the Health Canada Tier system and other nutrient profiling models worldwide, key features suggested to be included in future Canadian nutrient profiling systems are: assessment of “positive” nutrients along with the “negative” nutrients already included, as well as calculating an overall continuous nutrient profile score. These additional features would enable the HCST to better capture product differences, prevent the majority of foods being categorized into Tiers 2–3 and would provide incentive for food industry to reformulate food products to meet the government Tier 1 food criteria. Currently, the HCST Tier System is unable to differentiate many of food products, for example the food items in the BNS Food Group “jello, dessert toppings and pudding mixes-commercial” meet the criteria to belong to Tier 2 and Tier 3 of the HCST and therefore any efforts by food industry for improving these foods may go unnoticed as they will still be categorized in the same Tier. These proposed changes are especially critical if the HCST is used as the underlying nutrient profiling system or basis for a future “healthy” food labeling system.

## Conclusions

The 2014 HCST Tier system is a useful tool for public health initiatives trying to ensure adherence to EWCFG recommendations, which are currently not being met by all children and adolescents. Nonetheless, it should be noted that this system is geared towards achieving DRI recommendations and is not a good indicator of obesity, which is consistent with previous studies indicating compliance with EWCFG does not necessarily assure reduced risk of obesity or chronic disease [45, 46]. Thus, use of an index more in line with those of the United Kingdom, FSANZ, and/or the United States with a more comprehensive food evaluation and overall score, may be more applicable for the Canadian population where obesity and other chronic diseases are of major public health concern. Public health nutrition professionals and policy makers can use this knowledge as well as the characteristics of compliers compared to non-compliers, in the development of nutritional programs and policies, as well as in future Canadian nutrient profiling systems.

## Additional file

Additional file 1:
**Table S1.** Weighted analysis of energy intake (percentage) within the food groups by 2014 Health Canada Surveillance Tool Tier System among Canadian children (2–11 years of age) and adolescents (12–18 years of age).^*^ **Table S2.** Weighted analysis of characteristics of compliers, intermediates, and non-compliers based on the percentage of energy from Tier 4 foods and “other” foods among Canadian adolescents (12–18 years)^*, †^. **Table S3.** Weighted analysis of nutrient intakes (density approach) [50] by compliers, intermediates, and non-compliers based on the percentage of energy consumed from Tier 4 foods and “other” foods among Canadian adolescent (12–18 years)^*^. **Figure S1.** Weighted age-stratified analysis of classification of foods as a percentage of servings based on the 2014 Health Canada Surveillance Tool Tier system^*,†^ among Canadian population of a) Boys and Girls ages 9 to 13 years, and b) Boys and Girls ages 14 to 18 years inclusive. (DOCX 49 kb)
