# Supplementary material for: Assessing the nutritional quality of diets of Canadian children and adolescents using the 2014 Health Canada Surveillance Tool Tier System
Source: BMC Public Health. 2016 May 10;16:381. doi: 10.1186/s12889-016-3038-5 (PMC4862040; doi:10.1186/s12889-016-3038-5)
Supplement: Additional file 1: — Table S1. Weighted analysis of energy intake (percentage) within the food groups by 2014 Health Canada Surveillance Tool Tier System among Canadian children (2–11 years of age) and adolescents (12–18 years of age).* Table S2. Weighted analysis of characteristics of compliers, intermediates, and non-compliers based on the percentage of energy from Tier 4 foods and “other” foods among Canadian adolescents (12–18 years)*, †. Table S3. Weighted analysis of nutrient intakes (density approach) [50] by compliers, intermediates, and non-compliers based on the percentage of energy consumed from Tier 4 foods and “other” foods among Canadian adolescent (12–18 years)*. Figure S1. Weighted age-stratified analysis of classification of foods as a percentage of servings based on the 2014 Health Canada Surveillance Tool Tier system*,† among Canadian population of a) Boys and Girls ages 9 to 13 years, and b) Boys and Girls ages 14 to 18 years inclusive. (DOCX 49 kb) [file 12889_2016_3038_MOESM1_ESM.docx]

**Table S1.** Weighted analysis of energy intake (percentage) within the food groups by 2014 Health Canada Surveillance Tool Tier System among Canadian children (2-11 years of age) and adolescents (12-18 years of age).^*^

|  |  | **% Energy within Food Group** | |
| --- | --- | --- | --- |
| **Food Group** | | **Children**  **(2-11 years)** | **Adolescents (12-18 years)** |
| Fruit Sub-Group | |  |  |
|  | Fruit, Other than Juice Tier 1 | 50 | 42 |
|  | Fruit, Other than Juice Tier 2 | 2 | 2 |
|  | Fruit, Other than Juice Tier 3 | 1 | 1 |
|  | Fruit, Other than Juice Tier 4 | 0 | 0 |
|  | Fruit, Juice Tier 1 | 0 | 0 |
|  | Fruit, Juice Tier 2 | 46 | 53 |
|  | Fruit, Juice Tier 3 | 2 | 2 |
|  | Fruit, Juice Tier 4 | 0 | 0 |
| Vegetable Sub-Group | |  |  |
|  | Dark Green Tier 1 | 3 | 3 |
|  | Dark Green Tier 2 | 0 | 0 |
|  | Dark Green Tier 3 | 0 | 0 |
|  | Dark Green Tier 4 | 0 | 0 |
|  | Deep Yellow or Orange Tier 1 | 5 | 3 |
|  | Deep Yellow or Orange Tier 2 | 0 | 0 |
|  | Deep Yellow or Orange Tier 3 | 0 | 0 |
|  | Deep Yellow or Orange Tier 4 | 0 | 0 |
|  | Potatoes Tier 1 | 12 | 11 |
|  | Potatoes Tier 2 | 7 | 4 |
|  | Potatoes Tier 3 | 21 | 22 |
|  | Potatoes Tier 4 | 27 | 32 |
|  | Other Vegetables Tier 1 | 18 | 18 |
|  | Other Vegetables Tier 2 | 3 | 2 |
|  | Other Vegetables Tier 3 | 3 | 3 |
|  | Other Vegetables Tier 4 | 0 | 1 |
|  | Vegetable Juice & Cocktail Tier 1 | 0 | 0 |
|  | Vegetable Juice & Cocktail Tier 2 | 0 | 0 |
|  | Vegetable Juice & Cocktail Tier 3 | 1 | 0 |
|  | Vegetable Juice & Cocktail Tier 4 | 0 | 0 |
| Fruit & Vegetable Food Group | |  |  |
|  | Fruit, Other than Juice Tier 1 | 28 | 18 |
|  | Fruit, Other than Juice Tier 2 | 1 | 1 |
|  | Fruit, Other than Juice Tier 3 | 0 | 0 |
|  | Fruit, Other than Juice Tier 4 | 0 | 0 |
|  | Fruit, Juice Tier 1 | 0 | 0 |
|  | Fruit, Juice Tier 2 | 25 | 22 |
|  | Fruit, Juice Tier 3 | 1 | 1 |
|  | Fruit, Juice Tier 4 | 0 | 0 |
|  | Dark Green Tier 1 | 1 | 2 |
|  | Dark Green Tier 2 | 0 | 0 |
|  | Dark Green Tier 3 | 0 | 0 |
|  | Dark Green Tier 4 | 0 | 0 |
|  | Deep Yellow or Orange Tier 1 | 2 | 2 |
|  | Deep Yellow or Orange Tier 2 | 0 | 0 |
|  | Deep Yellow or Orange Tier 3 | 0 | 0 |
|  | Deep Yellow or Orange Tier 4 | 0 | 0 |
|  | Potatoes Tier 1 | 6 | 6 |
|  | Potatoes Tier 2 | 3 | 2 |
|  | Potatoes Tier 3 | 9 | 13 |
|  | Potatoes Tier 4 | 12 | 18 |
|  | Other Vegetables Tier 1 | 8 | 10 |
|  | Other Vegetables Tier 2 | 1 | 1 |
|  | Other Vegetables Tier 3 | 1 | 2 |
|  | Other Vegetables Tier 4 | 0 | 1 |
|  | Vegetable Juice & Cocktail Tier 1 | 0 | 0 |
|  | Vegetable Juice & Cocktail Tier 2 | 0 | 0 |
|  | Vegetable Juice & Cocktail Tier 3 | 0 | 0 |
|  | Vegetable Juice & Cocktail Tier 4 | 0 | 0 |
| Grain Products Food Group | |  |  |
|  | Whole Grain Tier 1 | 2 | 1 |
|  | Whole Grain Tier 2 | 7 | 7 |
|  | Whole Grain Tier 3 | 3 | 3 |
|  | Whole Grain Tier 4 | 2 | 2 |
|  | Non Whole Grain, Enriched Tier 1 | 14 | 17 |
|  | Non Whole Grain, Enriched Tier 2 | 23 | 27 |
|  | Non Whole Grain, Enriched Tier 3 | 18 | 13 |
|  | Non Whole Grain, Enriched Tier 4 | 20 | 18 |
|  | Non Whole Grain, Not Enriched Tier 1 | 0 | 0 |
|  | Non Whole Grain, Not Enriched Tier 2 | 6 | 7 |
|  | Non Whole Grain, Not Enriched Tier 3 | 3 | 2 |
|  | Non Whole Grain, Not Enriched Tier 4 | 3 | 3 |
| Milk & Alternatives Food Group | |  |  |
|  | Fluid Milk & Fortified Soy-Based Beverages Tier 1 | 15 | 22 |
|  | Fluid Milk & Fortified Soy-Based Beverages Tier 2 | 45 | 45 |
|  | Fluid Milk & Fortified Soy-Based Beverages Tier 3 | 36 | 26 |
|  | Fluid Milk & Fortified Soy-Based Beverages Tier 4 | 4 | 5 |
|  | Other Milk Alternatives Tier 1 | 0 | 0 |
|  | Other Milk Alternatives Tier 2 | 1 | 2 |
|  | Other Milk Alternatives Tier 3 | 0 | 0 |
|  | Other Milk Alternatives Tier 4 | 0 | 0 |
| Meat Sub-Group | |  |  |
|  | Beef, Game and Organ Meats Tier 1 | 0 | 0 |
|  | Beef, Game and Organ Meats Tier 2 | 2 | 3 |
|  | Beef, Game and Organ Meats Tier 3 | 26 | 30 |
|  | Beef, Game and Organ Meats Tier 4 | 0 | 0 |
|  | Other Meats (Pork, Veal, Lamb) Tier 1 | 0 | 0 |
|  | Other Meats (Pork, Veal, Lamb) Tier 2 | 0 | 0 |
|  | Other Meats (Pork, Veal, Lamb) Tier 3 | 10 | 9 |
|  | Other Meats (Pork, Veal, Lamb) Tier 4 | 0 | 0 |
|  | Poultry Tier 1 | 3 | 5 |
|  | Poultry Tier 2 | 5 | 6 |
|  | Poultry Tier 3 | 14 | 14 |
|  | Poultry Tier 4 | 9 | 5 |
|  | Processed Meats Tier 1 | 0 | 0 |
|  | Processed Meats Tier 2 | 0 | 1 |
|  | Processed Meats Tier 3 | 8 | 7 |
|  | Processed Meats Tier 4 | 23 | 18 |
| Meat Alternatives Sub-Group | |  |  |
|  | Fish Tier 1 | 4 | 4 |
|  | Fish Tier 2 | 6 | 6 |
|  | Fish Tier 3 | 1 | 1 |
|  | Fish Tier 4 | 5 | 3 |
|  | Shellfish Tier 1 | 1 | 1 |
|  | Shellfish Tier 2 | 2 | 1 |
|  | Shellfish Tier 3 | 1 | 1 |
|  | Shellfish Tier 4 | 0 | 1 |
|  | Legumes Tier 1 | 7 | 3 |
|  | Legumes Tier 2 | 0 | 1 |
|  | Legumes Tier 3 | 33 | 35 |
|  | Legumes Tier 4 | 2 | 2 |
|  | Nuts and Seeds Tier 1 | 0 | 0 |
|  | Nuts and Seeds Tier 2 | 1 | 2 |
|  | Nuts and Seeds Tier 3 | 8 | 9 |
|  | Nuts and Seeds Tier 4 | 2 | 4 |
|  | Eggs Tier 1 | 0 | 0 |
|  | Eggs Tier 2 | 26 | 24 |
|  | Eggs Tier 3 | 1 | 1 |
|  | Eggs Tier 4 | 0 | 0 |
| Meat & Alternatives Food Group | |  |  |
|  | Beef, Game and Organ Meats Tier 1 | 0 | 0 |
|  | Beef, Game and Organ Meats Tier 2 | 1 | 3 |
|  | Beef, Game and Organ Meats Tier 3 | 19 | 23 |
|  | Beef, Game and Organ Meats Tier 4 | 0 | 0 |
|  | Other Meats (Pork, Veal, Lamb) Tier 1 | 0 | 0 |
|  | Other Meats (Pork, Veal, Lamb) Tier 2 | 0 | 0 |
|  | Other Meats (Pork, Veal, Lamb) Tier 3 | 7 | 7 |
|  | Other Meats (Pork, Veal, Lamb) Tier 4 | 0 | 0 |
|  | Poultry Tier 1 | 2 | 4 |
|  | Poultry Tier 2 | 4 | 4 |
|  | Poultry Tier 3 | 10 | 11 |
|  | Poultry Tier 4 | 7 | 4 |
|  | Fish Tier 1 | 1 | 1 |
|  | Fish Tier 2 | 1 | 1 |
|  | Fish Tier 3 | 0 | 0 |
|  | Fish Tier 4 | 1 | 1 |
|  | Shellfish Tier 1 | 0 | 0 |
|  | Shellfish Tier 2 | 0 | 0 |
|  | Shellfish Tier 3 | 0 | 0 |
|  | Shellfish Tier 4 | 0 | 0 |
|  | Legumes Tier 1 | 2 | 1 |
|  | Legumes Tier 2 | 0 | 0 |
|  | Legumes Tier 3 | 8 | 8 |
|  | Legumes Tier 4 | 1 | 1 |
|  | Nuts and Seeds Tier 1 | 0 | 0 |
|  | Nuts and Seeds Tier 2 | 0 | 1 |
|  | Nuts and Seeds Tier 3 | 2 | 2 |
|  | Nuts and Seeds Tier 4 | 0 | 1 |
|  | Eggs Tier 1 | 0 | 0 |
|  | Eggs Tier 2 | 7 | 5 |
|  | Eggs Tier 3 | 0 | 0 |
|  | Eggs Tier 4 | 0 | 0 |
|  | Processed Meats Tier 1 | 0 | 0 |
|  | Processed Meats Tier 2 | 0 | 0 |
|  | Processed Meats Tier 3 | 6 | 6 |
|  | Processed Meats Tier 4 | 17 | 14 |
| Total Energy Intake | | N/A | N/A |

^*^Tiers are based on Health Canada’s Surveillance Tool [[19](#_ENREF_19)] and defined generally as follows: Tier 1-3 foods are compliant with EWCFG and Tier 4 foods are not recommended by the EWCFG. Tier 1 are foods that do not exceed lower thresholds for total fat, sugars, and sodium; Tier 2 foods do not exceed up to 2 lower thresholds for total fat, sugars or sodium, without exceeding any upper thresholds; for the Vegetables and Fruit and Grain Products food groups Tier 3 are foods that exceed all 3 lower thresholds without exceeding any upper thresholds or exceed only one upper threshold, while Tier 4 foods exceed at least 2 upper thresholds for total fat, saturated fat, sugars, or sodium. Within the Milk and Alternatives and Meat and Alternatives food groups, Tier 3 foods exceed all 3 lower thresholds without exceeding any upper thresholds for total fat, sugars, or sodium (irrespective of saturated fat) or exceed only one of these 3 thresholds or foods that only exceed the upper saturated fat threshold; within these 2 food groups foods that exceed at least 2 upper thresholds for total fat, sugars, or sodium were classified as Tier 4. Where lower thresholds entail: total fat < 3 g/RA, sugars < 6 g/RA, and sodium <140 mg/RA; and upper thresholds are: total fat >10 g/RA, sugars >19 g/RA, sodium >360 mg/RA, and saturated fat >2 g/RA.

**Table S2.** Weighted analysis of characteristics of compliers, intermediates, and non-compliers based on the percentage of energy from Tier 4 foods and “other” foods among Canadian adolescents (12-18 years)^*, †^

|  | | **Compliers (Q1)**^‡^  **≤24.69% Energy** | | **Intermediates (Q2)**^§^  **24.69-37.48% Energy** | | **Intermediates (Q3)**^§^  **37.48-51.68% Energy** | | **Non-compliers (Q4)**^‖^  **>51.68% Energy** | |  |
| --- | --- | --- | --- | --- | --- | --- | --- | --- | --- | --- |
| **Characteristics** | | **Mean** | **SEM** | **Mean** | **SEM** | **Mean** | **SEM** | **Mean** | **SEM** | **P-Trend** |
| Age (years) | | 15.04 | 0.16 | 14.79 | 0.13 | 14.69 | 0.16 | 15.13 | 0.16 | 0.0066 |
| Sex | |  |  |  |  |  |  |  |  |  |
|  | Males (%) | 46.85 | 3.05 | 55.16 | 2.75 | 55.00 | 3.56 | 50.21 | 2.36 |  |
|  | Females (%) | 53.15 | 3.05 | 44.84 | 2.75 | 45.00 | 3.56 | 49.79 | 2.36 | 0.0762 |
| BMI (kg/m^2^) | | 22.43 | 0.20 | 22.45 | 0.26 | 22.17 | 0.22 | 22.43 | 0.29 | 0.7795 |
| Reporters | |  |  |  |  |  |  |  |  |  |
|  | Under Reporters (%) | 60.71 | 2.38 | 65.51 | 2.43 | 56.57 | 2.70 | 53.14 | 2.53 |  |
|  | Plausible Reporters (%) | 60.71 | 2.38 | 65.51 | 2.43 | 56.57 | 2.70 | 53.14 | 2.53 |  |
|  | Over Reporters (%) | 14.90 | 1.90 | 16.77 | 2.04 | 24.94 | 2.49 | 28.56 | 2.47 | <.0001 |
| Physical Activity | |  |  |  |  |  |  |  |  |  |
|  | Inactive (%) | 32.08 | 2.56 | 35.88 | 2.54 | 36.41 | 2.88 | 30.86 | 2.33 |  |
|  | Moderate (%) | 25.61 | 2.14 | 23.85 | 2.23 | 24.57 | 2.46 | 28.69 | 2.43 |  |
|  | Active (%) | 42.31 | 2.60 | 40.27 | 2.44 | 39.03 | 2.58 | 40.44 | 2.59 | 0.5833 |
| Smoking Status | |  |  |  |  |  |  |  |  |  |
|  | Daily Smoker (%) | 1.90 | 0.55 | 4.18 | 0.94 | 4.36 | 0.87 | 5.46 | 1.02 |  |
|  | Occasional Smoker (%) | 1.57 | 0.55 | 2.10 | 0.56 | 3.57 | 0.89 | 5.63 | 1.51 |  |
|  | Former Smoker (%) | 1.03 | 0.48 | 1.88 | 0.62 | 1.29 | 0.50 | 2.51 | 0.72 |  |
|  | Never Smoked (%) | 95.48 | 1.01 | 91.83 | 1.31 | 90.77 | 1.33 | 86.33 | 2.01 | 0.0019 |

Abbreviation: SEM: Standard Error of Mean

^*^Adjusted for age and sex.

^†^Quartiles are based upon percentage of energy from all Tier 4 foods based on Health Canada’s Surveillance Tool Tier system 2014 plus “other” foods and beverages not recommended in the Eating Well with Canada’s Food Guide

^‡^The 25% of individuals with the lowest percentage of energy from Tier 4 and “other” foods.

^§^The individuals in the interquartile range for energy intakes from Tier 4 and “other” foods.

^‖^The 25% of individuals with the highest percentage of energy from Tier 4 and “other” foods

**Table S3.** Weighted analysis of nutrient intakes (density approach) [[50](#_ENREF_50)] by compliers, intermediates, and non-compliers based on the percentage of energy consumed from Tier 4 foods and “other” foods among Canadian adolescent (12-18 years)^*^

|  |  | **Compliers (Q1)**^†^  **≤24.69% Energy** | | **Intermediates (Q2)**^‡^  **24.69-37.48% Energy** | | **Intermediates (Q3)**^‡^  **37.48-51.68% Energy** | | **Non-compliers (Q4)**^§^  **>51.68% Energy** | | | |  |  |  |
| --- | --- | --- | --- | --- | --- | --- | --- | --- | --- | --- | --- | --- | --- | --- |
| **Nutrients** |  | **Mean** | **SEM** | **Mean** | **SEM** | **Mean** | **SEM** | **Mean** | **SEM** |  | **P-Trend** | | | |
| Energy (kcal/day) | a | 2251 | 60 | 2419 | 44 | 2540 | 60 | 2680 | 67 |  | <.0001 | | | |
|  | b | 2439.58 | 43.99 | 2508.93 | 32.76 | 2512.39 | 37.88 | 2595.60 | 40.25 |  | 0.04 | | | |
| Fat (%Energy) | a | 28.35 | 0.43 | 30.82 | 0.37 | 32.13 | 0.34 | 33.00 | 0.42 |  | <.0001 | | | |
|  | b | 28.52 | 0.43 | 30.91 | 0.40 | 32.11 | 0.35 | 32.93 | 0.44 |  | <.0001 | | | |
| Saturated fat (%Energy) | a | 10.80 | 0.20 | 12.06 | 0.19 | 12.80 | 0.16 | 13.13 | 0.20 |  | <.0001 | | | |
|  | b | 9.88 | 0.22 | 10.40 | 0.17 | 10.86 | 0.24 | 11.02 | 0.22 |  | 0.00 | | | |
| Monounsaturated fat (%Energy) | a | 10.80 | 0.20 | 12.06 | 0.19 | 12.80 | 0.16 | 13.13 | 0.20 |  | <.0001 | | | |
|  | b | 10.86 | 0.21 | 12.08 | 0.20 | 12.79 | 0.17 | 13.10 | 0.21 |  | <.0001 | | | |
| Polyunsaturated fat (%Energy) | a | 4.79 | 0.15 | 5.13 | 0.10 | 5.34 | 0.10 | 5.70 | 0.14 |  | <.0001 | | | |
|  | b | 4.85 | 0.16 | 5.18 | 0.11 | 5.37 | 0.10 | 5.72 | 0.15 |  | 0.00 | | | |
| Carbohydrates (%Energy) | a | 54.15 | 0.50 | 53.57 | 0.44 | 53.92 | 0.41 | 54.39 | 0.57 |  | 0.68 | | | |
|  | b | 53.87 | 0.51 | 53.35 | 0.47 | 53.84 | 0.42 | 54.37 | 0.59 |  | 0.55 | | | |
| Added sugar (%Energy) | a | 9.68 | 0.40 | 12.91 | 0.38 | 16.39 | 0.48 | 19.81 | 0.63 |  | <.0001 | | | |
|  | b | 9.65 | 0.42 | 12.90 | 0.39 | 16.41 | 0.51 | 19.84 | 0.63 |  | <.0001 | | | |
| Dietary fiber (g/1000 kcal) | a | 8.24 | 0.17 | 6.83 | 0.13 | 6.17 | 0.11 | 5.61 | 0.12 |  | <.0001 | | | |
|  | b | 8.21 | 0.17 | 6.82 | 0.14 | 6.18 | 0.11 | 5.64 | 0.13 |  | <.0001 | | | |
| Protein (%Energy) | a | 17.49 | 0.28 | 15.43 | 0.32 | 13.45 | 0.19 | 11.44 | 0.20 |  | <.0001 | | | |
|  | b | 17.50 | 0.28 | 15.48 | 0.30 | 13.51 | 0.20 | 11.51 | 0.21 |  | <.0001 | | | |
| Alcohol (%Energy) | a | 0.01 | 0.05 | 0.17 | 0.05 | 0.05 | 0.10 | 1.17 | 0.31 |  | <.0001 | | | |
|  | b | 0.11 | 0.08 | 0.26 | 0.08 | 0.54 | 0.11 | 1.19 | 0.31 |  | <.0001 | | | |
| Vitamin A (RE/1000 kcal) | a | 338.44 | 12.36 | 291.76 | 12.14 | 251.21 | 10.61 | 220.37 | 8.55 |  | <.0001 | | | |
|  | b | 337.41 | 12.81 | 292.25 | 11.93 | 252.74 | 10.41 | 222.46 | 8.61 |  | <.0001 | | | |
| Vitamin D (ug/1000 kcal) | a | 3.23 | 0.10 | 2.91 | 0.11 | 2.33 | 0.10 | 2.10 | 0.10 |  | <.0001 | | | |
|  | b | 3.25 | 0.10 | 2.94 | 0.11 | 2.35 | 0.10 | 2.13 | 0.10 |  | <.0001 | | | |
| Thiamin (mg/1000 kcal) | a | 1.01 | 0.02 | 0.90 | 0.02 | 0.78 | 0.01 | 0.61 | 0.01 |  | <.0001 | | | |
|  | b | 1.01 | 0.02 | 0.89 | 0.02 | 0.78 | 0.02 | 0.61 | 0.01 |  | <.0001 | | | |
| Riboflavin (mg/1000 kcal) | a | 1.11 | 0.02 | 1.01 | 0.02 | 0.93 | 0.04 | 0.77 | 0.01 |  | <.0001 | | | |
|  | b | 1.10 | 0.02 | 1.00 | 0.02 | 0.93 | 0.04 | 0.77 | 0.02 |  | <.0001 | | | |
| Niacin (NE/1000 kcal) | a | 19.70 | 0.31 | 17.48 | 0.39 | 15.27 | 0.18 | 13.06 | 0.23 |  | <.0001 | | | |
|  | b | 19.61 | 0.31 | 17.43 | 0.37 | 15.28 | 0.20 | 13.09 | 0.24 |  | <.0001 | | | |
| Vitamin B6 (ug/1000 kcal) | a | 0.95 | 0.02 | 0.82 | 0.02 | 0.67 | 0.01 | 0.56 | 0.01 |  | <.0001 | | | |
|  | b | 0.94 | 0.02 | 0.82 | 0.02 | 0.67 | 0.01 | 0.56 | 0.01 |  | <.0001 | | | |
| Folate (ug/1000 kcal) | a | 111.93 | 2.99 | 95.80 | 2.46 | 82.31 | 2.01 | 70.52 | 2.25 |  | <.0001 | | | |
|  | b | 110.55 | 2.96 | 94.93 | 2.59 | 82.21 | 2.19 | 70.78 | 2.36 |  | <.0001 | | | |
| Vitamin B12 (ug/1000 kcal) | a | 2.18 | 0.07 | 1.92 | 0.08 | 1.59 | 0.05 | 1.37 | 0.06 |  | <.0001 | | | |
|  | b | 2.19 | 0.08 | 1.94 | 0.07 | 1.61 | 0.06 | 1.38 | 0.07 |  | <.0001 | | | |
| Vitamin C (mg/1000 kcal) | a | 79.65 | 3.20 | 71.18 | 3.41 | 65.60 | 3.25 | 50.96 | 2.26 |  | <.0001 | | | |
|  | b | 77.62 | 3.21 | 69.81 | 3.58 | 65.32 | 3.49 | 51.18 | 2.31 |  | <.0001 | | | |
| Calcium (mg/1000 kcal) | a | 553.15 | 12.83 | 482.20 | 12.10 | 433.91 | 12.71 | 374.93 | 8.01 |  | <.0001 | | | |
|  | b | 557.30 | 12.83 | 488.04 | 12.28 | 438.78 | 13.10 | 379.53 | 8.60 |  | <.0001 | | | |
| Phosphorous (mg/1000 kcal) | a | 727.40 | 9.78 | 636.49 | 9.20 | 572.58 | 8.88 | 505.76 | 7.84 |  | <.0001 | | | |
|  | b | 727.81 | 9.69 | 638.14 | 9.38 | 574.57 | 9.28 | 508.00 | 8.19 |  | <.0001 | | | |
| Potassium (mg/1000 kcal) | a | 1568.27 | 25.46 | 1366.81 | 20.67 | 1191.45 | 15.74 | 1049.93 | 18.77 |  | <.0001 | | | |
|  | b | 1557.19 | 25.46 | 1360.71 | 22.08 | 1191.87 | 17.48 | 1053.48 | 19.16 |  | <.0001 | | | |
| Sodium (mg/1000 kcal) | a | 1530.75 | 33.05 | 1462.75 | 23.38 | 1441.09 | 22.31 | 1389.53 | 25.26 |  | 0.01 | | | |
|  | b | 1521.63 | 31.44 | 1459.61 | 25.02 | 1444.09 | 23.31 | 1395.59 | 26.51 |  | 0.03 | | | |
| Magnesium (mg/1000 kcal) | a | 158.65 | 2.03 | 133.65 | 1.88 | 120.11 | 1.43 | 106.81 | 1.58 |  | <.0001 | | | |
|  | b | 157.79 | 2.02 | 133.36 | 1.88 | 120.40 | 1.55 | 107.39 | 1.59 |  | <.0001 | | | |
| Iron (mg/1000 kcal) | a | 7.79 | 0.12 | 7.21 | 0.24 | 6.35 | 0.12 | 5.29 | 0.07 |  | <.0001 | | | |
|  | b | 7.83 | 0.14 | 7.29 | 0.28 | 6.42 | 0.14 | 5.36 | 0.09 |  | <.0001 | | | |
| Zinc (mg/1000 kcal) | a | 5.78 | 0.11 | 5.15 | 0.09 | 4.52 | 0.08 | 3.86 | 0.07 |  | <.0001 | | | |
|  | b | 5.77 | 0.11 | 5.14 | 0.09 | 4.52 | 0.08 | 3.86 | 0.07 |  | <.0001 | | | |
| Glycemic Index | a | 52.93 | 0.30 | 55.47 | 0.31 | 57.25 | 0.31 | 58.10 | 0.38 |  | <.0001 | | | |
|  | b | 52.77 | 0.31 | 55.33 | 0.32 | 57.18 | 0.33 | 58.06 | 0.39 |  | <.0001 | | | |
| Glycemic Load | a | 163.78 | 4.30 | 178.84 | 3.63 | 198.00 | 4.76 | 212.46 | 5.05 |  | <.0001 | | | |
|  | b | 176.13 | 3.58 | 183.85 | 2.90 | 195.01 | 3.44 | 205.53 | 3.70 |  | <.0001 | | | |
| Energy Density (kcal/g) | a | 1.80 | 0.03 | 2.06 | 0.03 | 2.24 | 0.03 | 2.43 | 0.03 |  | <.0001 | | | |
|  | b | 1.82 | 0.03 | 2.07 | 0.03 | 2.24 | 0.03 | 2.43 | 0.03 |  | <.0001 | | | |

Abbreviation: SEM: Standard Error of Mean

a Means are adjusted for age and sex.

b Means are adjusted for age, sex, and misreporting status (under-reporter, plausible-, and over-reporters)

^*^Quartiles are based upon percentage of energy from all Tier 4 foods based on Health Canada’s Surveillance Tool Tier system 2014 plus “other” foods and beverages not recommended in the Eating Well with Canada’s Food Guide

^†^The 25% of individuals with the lowest percentage of energy from Tier 4 and “other” foods.

^‡^The individuals in the interquartile range for energy intakes from Tier 4 and “other” foods.

^§^The 25% of individuals with the highest percentage of energy from Tier 4 and “other” foods

**Figure S1.** Weighted age-stratified analysis of classification of foods as a percentage of servings based on the 2014 Health Canada Surveillance Tool Tier system^*,†^ among Canadian population of a) Boys and Girls ages 9 to 13 years, and b) Boys and Girls ages 14 to 18 years inclusive.

^*^Energy adjusted.

^†^Tiers are based on Health Canada’s Surveillance Tool [[19](#_ENREF_19)] and defined generally as follows: Tier 1-3 foods are compliant with EWCFG and Tier 4 foods are not recommended by the EWCFG. Tier 1 are foods that do not exceed lower thresholds for total fat, sugars, and sodium; Tier 2 foods do not exceed up to 2 lower thresholds for total fat, sugars or sodium, without exceeding any upper thresholds; for the Vegetables and Fruit and Grain Products food groups Tier 3 are foods that exceed all 3 lower thresholds without exceeding any upper thresholds or exceed only one upper threshold, while Tier 4 foods exceed at least 2 upper thresholds for total fat, saturated fat, sugars, or sodium. Within the Milk and Alternatives and Meat and Alternatives food groups, Tier 3 foods exceed all 3 lower thresholds without exceeding any upper thresholds for total fat, sugars, or sodium (irrespective of saturated fat) or exceed only one of these 3 thresholds or foods that only exceed the upper saturated fat threshold; within these 2 food groups foods that exceed at least 2 upper thresholds for total fat, sugars, or sodium were classified as Tier 4. Where lower thresholds entail: total fat < 3 g/RA, sugars < 6 g/RA, and sodium <140 mg/RA; and upper thresholds are: total fat >10 g/RA, sugars >19 g/RA, sodium >360 mg/RA, and saturated fat >2 g/RA.

**Supplementary Figure 1.**

a)

b)
